# Metabolomic Investigations on *Nesterenkonia flava* Revealed Significant Differences between Marine and Terrestrial Actinomycetes

**DOI:** 10.3390/md16100356

**Published:** 2018-09-30

**Authors:** Chun-Lan Xie, Jin-Mei Xia, Jun-Song Wang, Dong-Hai Lin, Xian-Wen Yang

**Affiliations:** 1 State Key Laboratory Breeding Base of Marine Genetic Resources, Fujian Key Laboratory of Marine Genetic Resources, South China Sea Bio-Resource Exploitation and Utilization Collaborative Innovation Center, Third Institute of Oceanography, State Oceanic Administration, 184 Daxue Road, Xiamen 361005, China; xiechunlanxx@163.com (C.-L.X.); xiajinmei@tio.org.cn (J.-M.X.); 2 College of Chemistry and Chemical Engineering, The Key Laboratory for Chemical Biology of Fujian Province, MOE Key Laboratory of Spectrochemical Analysis & Instrumentation, Xiamen University, 422 Siming South Road, Xiamen 361005, China; 3 Center for Molecular Metabolism, School of Environmental and Biological Engineering, Nanjing University of Science and Technology, 200 Xiaolingwei Street, Nanjing 210094, China

**Keywords:** microorganism, metabolism, environment

## Abstract

Marine microorganisms are an important source of natural products with potent bioactivities. Unlike the land, the ocean, especially the deep-sea, is characterized by high pressure, high salinity, low nutrition, and no light among others. Therefore, the biodiversity of marine microorganisms is supposed to be very different from that of the terrestrial ones. Yet, many marine microorganisms can find their counterparts in terrestrial environments. To evaluate their differences, a comparative metabolomics investigation was performed on four strains of *Nesterenkonia flava* isolated from terrestrial and marine environments. As a result, marine strains were clearly distinguished from terrestrial ones on the principal components analysis (PCA) score plot. Furthermore, by partial least squares discrimination analysis (PLS-DA) and univariate analysis, the characteristic metabolites were figured out and found to be involved in osmotic regulation, redox balancing, and energy metabolism. Our results demonstrated that marine actinomycetes could produce novel secondary metabolites different from their terrestrial relatives because they have special metabolic patterns closely related to the unique features of their living environment.

## 1. Introduction

During the past few decades, natural products have played a dominant role in discovery of drugs for the treatment of various diseases [1]. Among diverse sources, secondary metabolites from microbes formed powerful driving forces of pharmaceutical discovery. Marine microorganisms, especially those from deep-sea origin, are believed to possess greater potential than terrestrial ones in generating diverse secondary metabolites with various bioactivities [2,3]. It is believed that adaptations of harsh environmental conditions, such as high pressure, low/high temperature, high saline etc., could lead marine microbes to produce structurally novel and biologically significant compounds [4]. Of all the microorganisms, actinomycetes are believed to be the richest sources of secondary metabolites, producing about two-thirds of naturally occurring antibiotics [5,6].

The ocean covers more than 70% of the Earth’s surface. It is believed that the biodiversity in the ocean is much more abundant than that of the terrestrial environment [7]. Although many interesting new compounds have been isolated from the highly complex and adapted marine microorganisms [8,9], considerable debates still exist about the relationship between marine and terrestrial microbes. Some claim that most of marine microbes occur exclusively in the sea [10], while others argue that marine microbes are actually from the land since spore-forming microorganisms in soils could be washed into the sea. These disagreements make it more difficult to evaluate to what extent marine microbes are evolutionarily, ecologically, or chemically distinct from their terrestrial relatives [11,12]. As an ideal tool, metabolomics can give comprehensive information on identification and quantification of microbial metabolites [13,14,15]. Previously, intraspecific comparative analyses were conducted on different terrestrial microbes [16]. However, there is no such investigation on marine and terrestrial microbes.

*Nesterenkonia* is an important genus of the *Micrococcaceae* family consisting of 12 species [17]. They are halophilic/halotolerant and alkaliphilic/alkalitolerant actinomycetes occurring around the world [18]. Herein, we report the intraspecies differences on marine and terrestrial *Nesterenkonia flava* using ^1^H nuclear magnetic resonance (NMR)-based metabolomics.

## 2. Results and Discussion

### 2.1. Comparison of Metabolite Profiles Between Marine and Terrestrial N. flava

**Figure 1:**
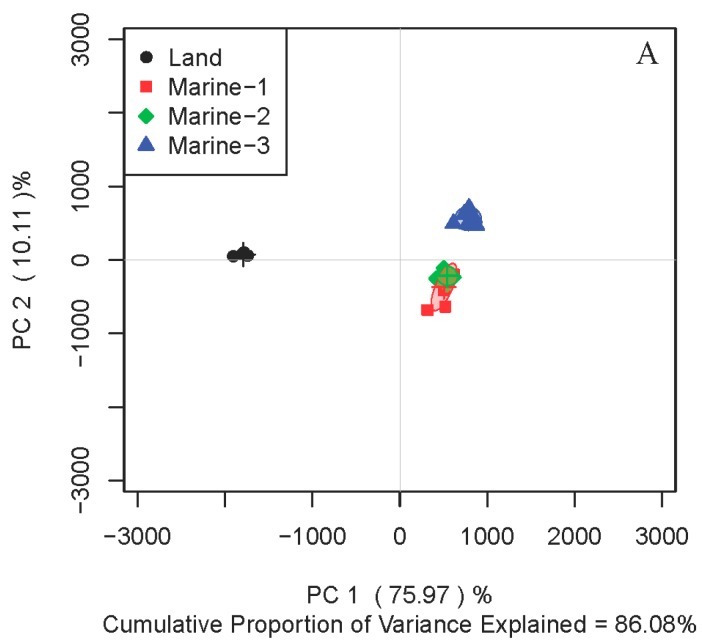

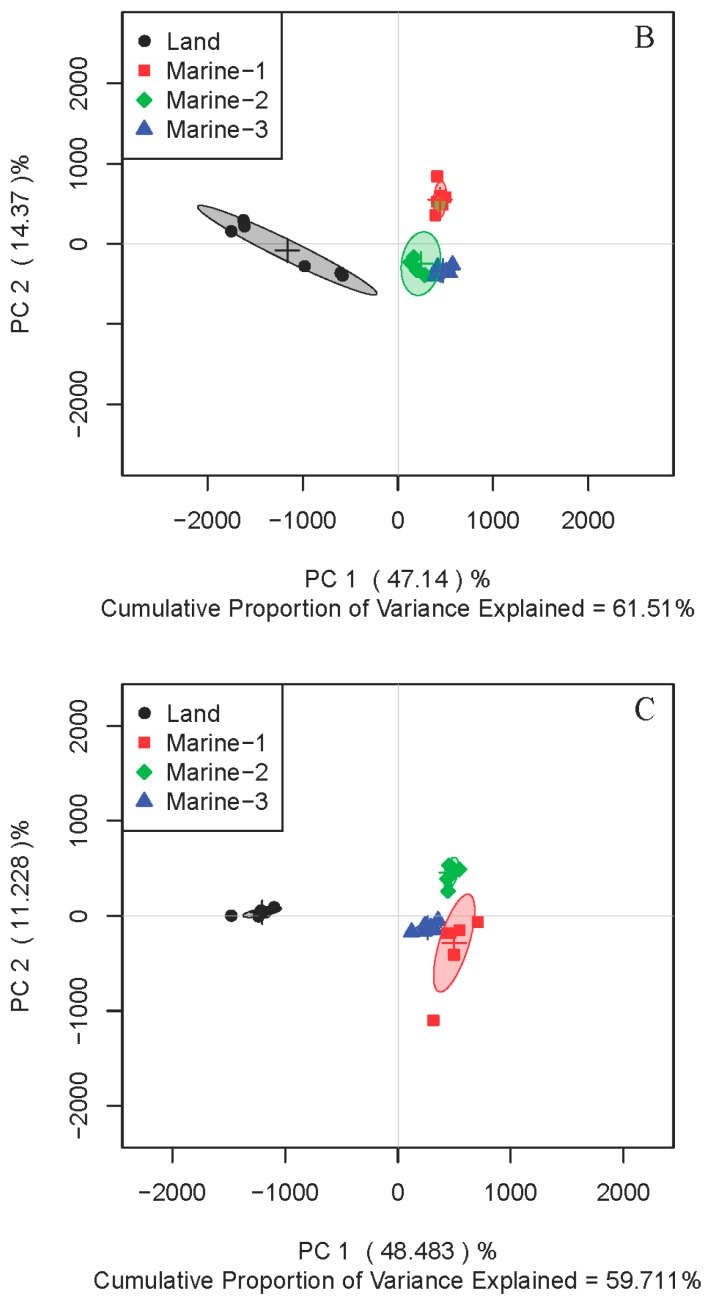
Principal components analysis (PCA) score plots of ^1^H nuclear magnetic resonance (NMR) data for intracellular metabolites extracted from four strains of *Nesterenkonia flava* in culture media M1 (**A**), M2 (**B**), and M3 (**C**).

To compare the metabolite profiles between strains of different origins, principal components analysis (PCA) of the intracellular metabolites were performed on four stains of *N. flava*, including one terrestrial (Land) and three marine strains (Marine-1–Marine-3). In medium M1, three marine strains were located together, discriminating from that of the terrestrial one on the first principal component of the score plot (Figure 1A). Since the metabolite profiles of microorganisms can be affected by the medium applied, another two different culture media were adopted for further investigation. Interestingly, the same trend was observed for media M2 (Figure 1B) and M3 (Figure 1C). Therefore, all three strains of marine *N. flava* in different culture media clustered together in the score plots, which were distinguished from the terrestrial strain.

**Figure 2:**
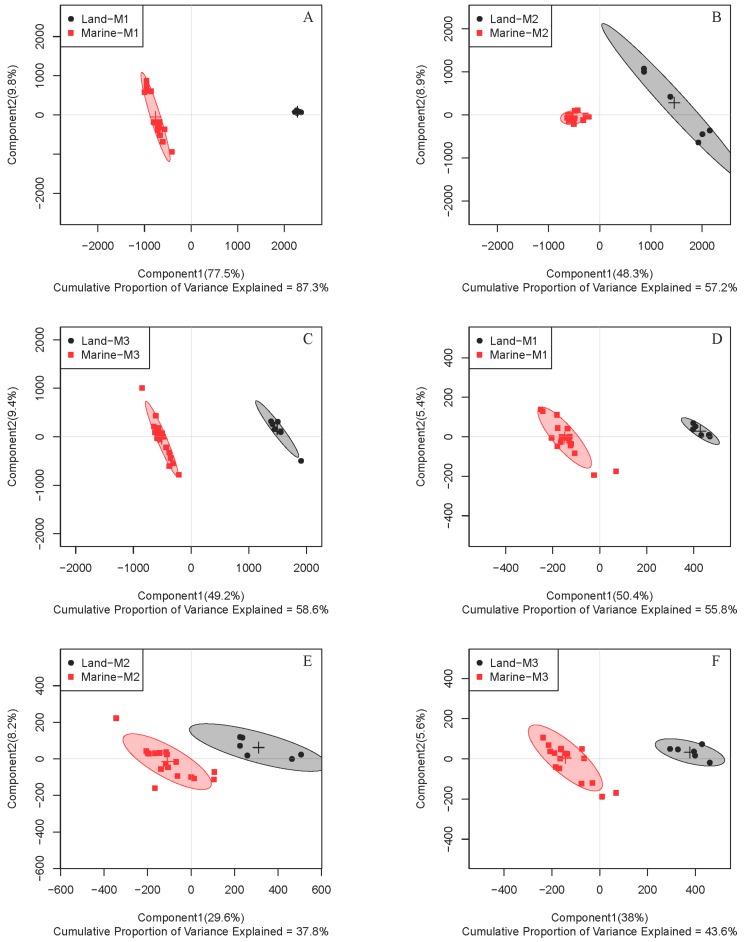
Partial least squares discrimination analysis (PLS-DA) score plots of ^1^H NMR data for intracellular (**A**–**C**) and extracellular (**D**–**F**) metabolites of four *N. flava* cultivated in media M1 (**A**,**D**), M2 (**B**,**E**), and M3 (**C**,**F**). The three marine stains of *N. flava* were treated as one group for all cases.

To further address the metabolic differences between marine and terrestrial *N. flava*, three strains of marine *N. flava* were treated as one group. Partial least squares discrimination analysis (PLS-DA) for both intracellular (primary) and extracellular (secondary) metabolites in three media showed clear discrimination between the two groups (Figure 2).

Since similar clustering trends were observed in all three studied media, samples from one medium can be used as a representative. Through intuitive observation, the metabolites are more diverse when using medium M3 and thus possess a better potential for further separation of novel compounds. So medium M3 was chosen for subsequent study. In order to understand the difference of metabolic patterns from the perspective of metabolic pathways, further analysis was focused on intracellular metabolites. From the PLS-DA score plot, an excellent distinction of intracellular metabolites was illustrated between the two groups in medium M3 (Figure 2C). A random permutation test (*n* = 200) was further conducted, and the parameters obtained affirmed the validity of the PLS-DA model (Figure S1).

**Figure 3:**
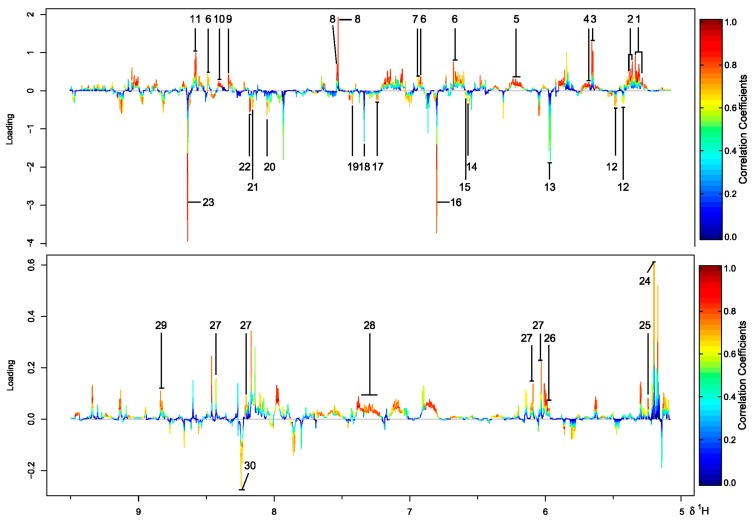
Color-coded loading plot from PLS-DA of ^1^H NMR data for four strains of *N. flava* cultivated in medium M3. The labeled metabolites: **1** Pantothenate, **2** Leucine, **3** 3-Hydroxybutyrate, **4** Fucose, **5** Lysine, **6** Glutamate, **7** Pyruvate, **8** Trimethylamine, **9** Indole-3-acetate, **10** Galactitol, **11** Mannitol, **12** Valine, **13** Alanine, **14** *N*-Acetylglucosamine, **15** *N*-Acetylcysteine, **16** *p*-Cresol, **17** Carnosine, **18** Sarcosine, **19** Methylguanidine, **20** Mannose, **21** Sucrose, **22** Arabinose, **23** Vanillate, **24** *N*-Acetyl-d-glucosamine, **25** Glucose, **26** UDP-glucose, **27** NAD^+^, **28** Cholate, **29** NADP^+^, **30** NADH.

Metabolite variations were then visualized using the loading plot, which was color coded according to the absolute correlation coefficient of each variable to grouping. Positive and negative peaks indicate a relatively higher or lower metabolite level in the terrestrial group, respectively. The signals with a warm color contribute more to class separation than those with a cold color. A total of 30 significant metabolites were found to be responsible for the difference. Among them, 13 were more abundant in the marine group (Figure 3). The loading plots for samples from medium M1 and M2 were shown in Figures S2 and S3.

### 2.2. Comparison of Metabolite Concentrations Between Marine and Terrestrial N. flava

**Figure 4:**
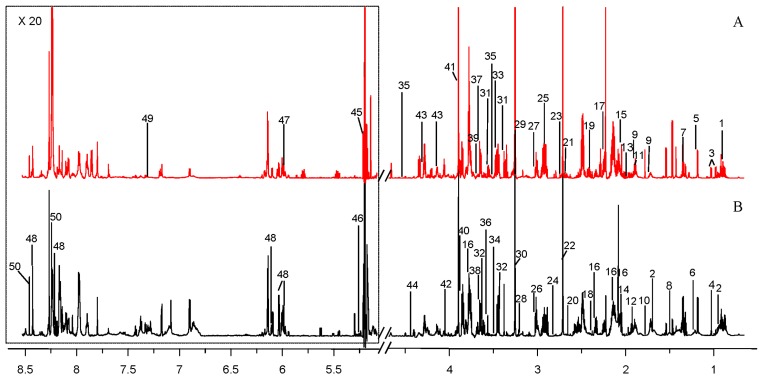
Representative ^1^H NMR (850 MHz) spectra of intracellular metabolites derived from marine *N. flava* MCCC 1K00610 (Marine-3, **A**) and terrestrial *N. flava* MCCC 1A10663 (Land, **B**) in medium M3. The spectral peaks were labeled with identified metabolites. The low field region framed in the square has been magnified 20 times. The labeled metabolites were listed in Table S1.

**Figure 5:**
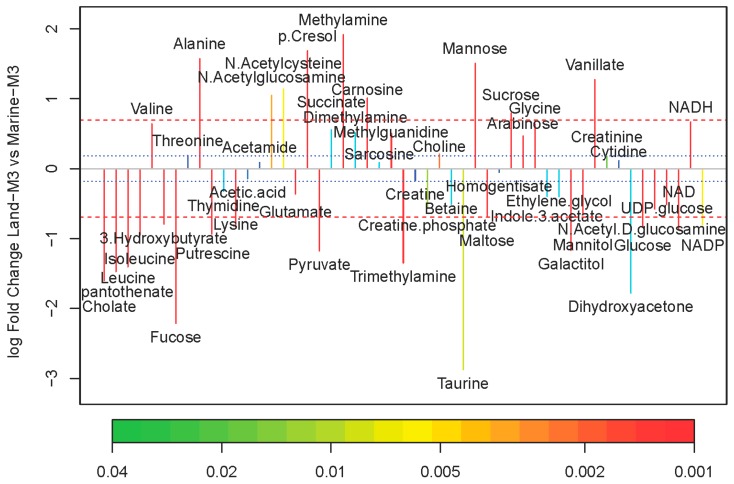
Fold change plot of metabolites color-coded with *p*-values adjusted by Benjamini–Hochberg method. Medium M3 was used for cultivation. Blue and red dashed lines denote variations of 20% and 100%, respectively.

The major metabolites were labeled in the representative ^1^H NMR (850 MHz) spectra for the strain Marine-3 and Land in medium M3 (Figure 4). Altogether, 50 metabolites were assigned (Table S1). The quantitative comparison of intracellular metabolites between the terrestrial and marine strains were visualized by a fold change plot (Figure 5). The upper section of the fold change plot represented metabolites that were more abundant in the marine strain compared with the terrestrial strains. The important differential metabolites were chosen according to the *p*-value in Student’s *t*-test (<0.05) and the fold change value (>1.5 or <0.67). Consequently, 34 differential metabolites were selected, 20 of which were more abundant in the terrestrial strain.

### 2.3. Inter-Metabolite Correlations for Marine and Terrestrial N. flava

**Figure 6:**
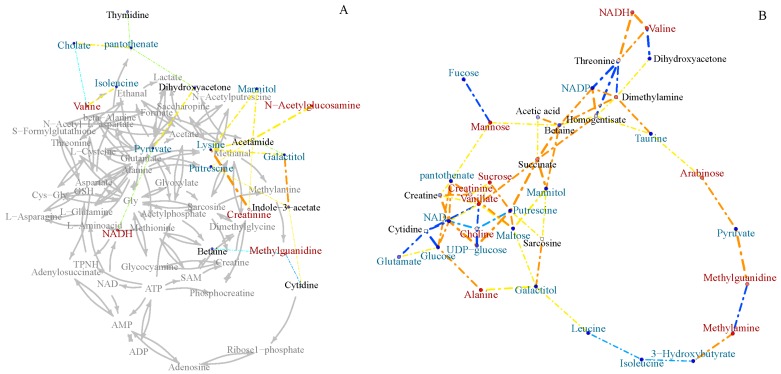
Pearson’s inter-metabolite correlation networks specific to marine (**A**) and terrestrial (**B**) *N. flava* in medium M3.

To further investigate the metabolic differences between marine and terrestrial *N. flava*, Pearson’s correlation networks were established on the 50 identified metabolites (Figure 6). As a result, more correlations (coefficients over 0.6) in the terrestrial group were found than that of the marine group. By close comparison, the correlations of betaine, mannitol, NAD^+^, NADP, NADH, glucose, pyruvate, and succinate with other metabolites varied greatly between two groups. Specifically, betaine is positively correlated with succinate, mannitol, NADP, dimethylamine, acetic acid, mannose, and homogentisate in the terrestrial strains, whereas it is negatively correlated with methylguanidine in the marine group.

Betaine and mannitol were reported to be compatible solutes. NAD^+^, NADP and NADH participate in redox reactions by exchanging electrons with other molecules. Glucose, pyruvate, and succinate are involved in glycolysis and the tricarboxylic acid (TCA) cycle. These results indicated that the two groups differed greatly in osmotic regulation, redox balancing, and energy metabolism.

### 2.4. Differential Metabolic Network Between Marine and Terrestrial N. flava

**Figure 7:**
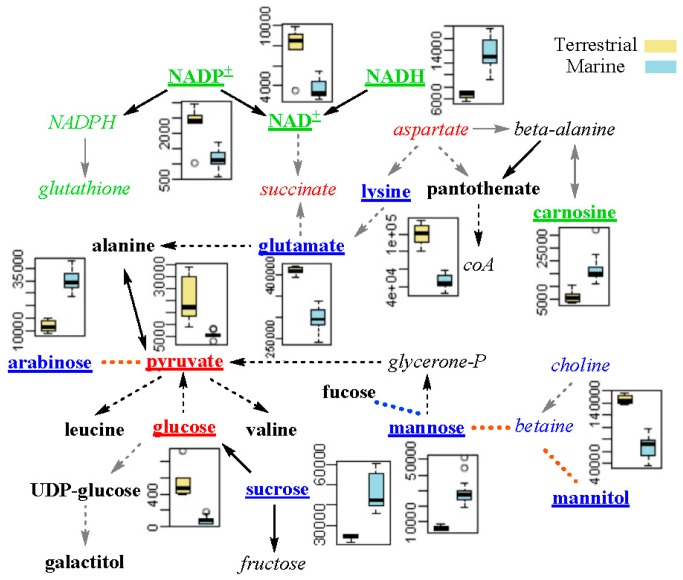
Schematic diagram of the metabolic differences between marine and terrestrial strains of *N. flava*. Metabolites of the blue, green, and red fonts were involved in osmotic regulation, redox balancing and energy metabolism, respectively. Metabolites in italics were not included in the 27 characteristic metabolites. The underlined metabolites were those with their boxplots shown. Yellow dashed lines denote positive inter-metabolites correlation. Solid and dashed arrows show one-step and multi-step chemical reactions, respectively. Grey arrows indicate missing of one or more enzymes for the chemical reactions in the Kyoto Encyclopedia of Genes and Genomes (KEGG) database.

A total of 27 characteristic metabolites were determined by taking the intersection of significant metabolites from PLS-DA and differential metabolites from univariate analysis (Table S2). Using the Pearson’s correlations, the differential metabolic network was then constructed. Consequently, the metabolites were found to be involved in osmotic regulation, redox balancing, and energy metabolism (Figure 7).

As is known, hyperosmotic shock caused by high salinity in the environment can result in a temporary loss of turgor pressure for microorganisms. As a response, they raise their internal compatible solute levels to increase internal osmotic pressure [19]. Salts (such as NaCl and KCl) and sugars (such as sucrose and mannose) are the most common solutes. Three kinds of sugars, mannose, sucrose, and arabinose were more abundant in marine *N. flava*. Lysine, glutamate, and mannitol were also reported to be osmotic regulation metabolites [20,21,22]. They were of higher concentration in the terrestrial *N. flava*. This result indicates that marine and terrestrial *N. flava* may have different osmotic adjustment substances.

According to previous study, high hydrostatic pressure can generate oxidative stress [23,24]. Carnosine was reported to scavenge reactive oxygen species (ROS) [25,26]. NADH is involved in redox reactions by donating electrons to other molecules, while NAD^+^ and NADP^+^ are the oxidized form of NADH and NADPH, respectively. In this work, NAD^+^ and NADP^+^ were of lower level, whereas NADH and carnosine were of higher level in the marine *N. flava*, which indicates that hydrostatic pressure could affect the capacity of microorganisms in coping with oxidative stress.

The energy metabolism of bacteria was more active in the eutrophic conditions compared to the oligotrophic environments, as indicated by faster utilization of glucose [27]. As two important energy compounds involved in glycolysis and TCA cycle, glucose and pyruvate were of lower levels in marine *N. flava*. This might be due to the lower nutrition level in the marine than in the terrestrial environments.

## 3. Materials and Methods

### 3.1. Bacterial Material

The terrestrial *Nesterenkonia flava* (Land) was isolated from the waste water of a paper mill in Wuhan, Hubei province, China [28]. Three marine strains of *Nesterenkonia flava* (Marine-1–Marine-3) were isolated from the eastern Pacific Ocean of −5098 m (W154°49.8′, N10°29.2′), −5368 m (W145°23.4′, N08°25.0′), and −5302 m (W145°21.7′, N08°26.6′′) [29]. The four strains of *Nesterenkonia flava* (Land, Marine-1, Marine-2 and Marine-3) were all deposited in Marine Culture Collection of China (MCCC) with the accession numbers 1A10663, 1K00606, 1K00607, and 1K00610, respectively.

### 3.2. Culture Media

Medium M1 (1 L, pH = 7.4) contains 15 g starch, 15 g glycerol, 5 g soy peptone, 15 g bacterial peptone, 30 g sea salt, and 2 g CaCO_3_. Medium M2 (1 L, pH = 7.2) contains 20 g starch, 10 g glucose, 5 g yeast extract powder, 5 g bacterial peptone, 30 g sea salt, and 5 g CaCO_3_. Medium M3 (1 L, pH = 7.2) contains 15 g glycerol, 7.5 g yeast extract powder, 7.5 g tryptone, and 15 g sea salt.

### 3.3. Bacteria Culture

The bacteria strains were cultivated on agar plates for 5 days. Then each strain was inoculated into a 250 mL Erlenmeyer flask containing 50 mL of medium to continue cultivation at 180 rpm under 28 °C for 48 h to reach exponential phase. After that, the strains were inoculated with an inoculum size of 5% into 6 new parallel Erlenmeyer flasks. They were then cultured for another 8 days.

### 3.4. Extraction of Metabolites

The broth cultures (50 mL) were harvested via centrifugation at 7000× *g* for 10 min under 4 °C. The supernatant was extracted by EtOAc and was freeze-dried as extracellular metabolites. The pellet was quenched using 10 mL of 60% cold MeOH (−80 °C) containing 0.85% (*w*/*v*) of NaCl for 30 min. The quenched cell pellets were re-suspended in 10 mL of cold PBS and were washed for 3 times. The mixture was then centrifuged at 9800× *g* under 4 °C for 5 min.

For each cell pellet sample, 5 mL of the mixture of MeOH-H_2_O (10:9, *v*/*v*) was added and ultrasonicated for 25 min on ice. The mixture was added with 5 mL of cold CHCl_3_, vortexed and subjected to 10 min of ultrasonic extraction. The mixture was then centrifuged at 9500× *g* for 8 min. The upper layer phase was taken out and freeze-dried to afford dry samples of intracellular metabolites.

### 3.5. ^1^H Nuclear Magnetic Resonance (NMR) Analysis of Samples

The metabolites were re-suspended in 550 µL of phosphate buffer containing 1.5 M KH_2_PO_4_, 0.1% sodium 3-(trimethylsilyl)propionate-2,2,3,3 (TSP)-*d*_4_, and 10% D_2_O. All samples were vortexed and centrifuged at 12,000× *g* for 15 min at 4 °C to remove any insoluble components. The collected supernatants (500 μL) were transferred to 5 mm NMR tubes for analysis.

All ^1^H NMR experiments were conducted on a Bruker Avance III 850 MHz spectrometer at 25 °C. Water suppression was achieved by irradiation of the water resonance during the recycle delay (RD) of 4 s with the mixing time (τm) of 120 ms. The spectral width was 10 kHz with an acquisition time per scan of 1.64 s, and 256 transients were collected into 32 K data points for each spectrum. The free induction decay (FID) was zero-filled to 64 K and an exponential line-broadening function of 0.3 Hz was applied to the FID before Fourier transformation.

### 3.6. Data Processing, Bioinformatics, and Statistical Analyses

All ^1^H NMR spectra were manually phased and corrected for baseline distortion, referenced to the methyl group of TSP at *δ*_H_ 0.00 and carefully aligned using Bruker Topspin 3.0 software (Bruker GmbH, Karlsruhe, Germany). The spectra were then converted to ASCII-format files and imported into “R” (http://cran.r-project.org/) and aligned with an in-house developed R-script. The ^1^H-NMR spectra were segmented into integrated regions with an average width of 0.015 ppm (bin) corresponding to the region of *δ* 0.65‒9.50 using R software. The region of *δ*_H_ 4.70‒5.10 was removed to eliminate artifacts related to the residual water resonance. The data were then probability quotient-normalized to compensate for variation in total sample volumes. Before multivariate statistical analysis, the integral values were mean-centered and Pareto-scaled. To check general separation and identify the outliers, PCA was performed on NMR data sets of all cell samples. PLS-DA was subsequently used to improve the separation. The validity of each PLS-DA model against overfitting was assessed using the parameter R^2^, and the predictive ability was described by Q^2^.

### 3.7. Identification of Intracellular Metabolites

Resonances of primary metabolites were assigned by querying publicly accessible metabolomics databases such as Human Metabolome Database (HMDB, http://www.hmdb.ca), Madison-Qingdao Metabolomics Consortium Database (MMCD, http://mmcd.nmrfam.wisc.edu/), and *E. coli* Metabolome Database (ECMDB, http://www.ecmdb.ca/). The assignments and integrations of peaks were aided by two-dimensional STOCSY, performed by a suite of in-house developed scripts running in “R”, and finally confirmed by 2D HSQC and TOCSY experiments.

### 3.8. Univariate Analysis

Univariate analysis was used to assess the integration area of metabolites over time and among groups using R software. The areas of metabolites were first tested for their conformity to the normality of the distribution. If the distribution followed the normality assumption, a parametric Student’s *t*-test was applied; otherwise, a non-parametric (Mann–Whitney test) test was performed to detect statistically significant metabolites. The threshold for significance was *p* < 0.05 for all tests. The fold change values of metabolites between groups were calculated and the associated *p*-values were adjusted by the Benjamini–Hochberg method [30] for controlling the false positive rate in multiple comparisons using scripts written in R language (http://stat.ethz.ch/R-manual/R-devel/library/stats/html/p.adjust.html).

### 3.9. Correlation Network Analysis

Pearson’s correlations among metabolites were calculated using R software, and the correlation networks were generated using igraph library package. Metabolites with coefficients of Pearson’s correlations greater than 0.6 were shown connected by dashed colored lines. The lines were coded according to the values of the coefficients with warm colors representing positive correlations and cool colors representing negative correlations. In addition, the linewidth was scaled based on the absolute values of the coefficients. The node circles were filled with colors from bluish to redish corresponding to the log_2_ (fold change) values of marine strains relative to the terrestrial strain. The gray arrows between the metabolites were used to indicate direct biological reactions.

## 4. Conclusions

Significant differences were observed for both extracellular and intracellular metabolic patterns between marine and terrestrial *N. flava*. Compared to their terrestrial counterpart, marine *N. flava* are of higher capacity in redox balancing, lower levels of energy metabolism, and different kinds of compatible solutes due to the high hydrostatic pressure, low nutrients, and high salinity of their living environments. These distinctions in primary metabolism are believed to be the underlying cause for the discrimination of their secondary metabolite profiles.

## Supplementary Materials

The following are available online at http://www.mdpi.com/1660-3397/16/10/356/s1, Figures S1–S3 and Tables S1 and S2: The PLS-DA scatter plots of statistical validation, the loading plots of samples from medium M1 and M2, ^1^H-NMR assignments of intracellular metabolites and the list of characteristic metabolites.
