# Supplementary material for: Metabolomic Investigations on Nesterenkonia flava Revealed Significant Differences between Marine and Terrestrial Actinomycetes
Source: Mar Drugs. 2018 Sep 30;16(10):356. doi: 10.3390/md16100356 (PMC6213030; doi:10.3390/md16100356)
Supplement: Supplementary file 1 [file marinedrugs-16-00356-s001.pdf]

Supplementary Data

Metabolomic Investigations on *Nesterenkonia flava* Revealed Significant Differences between Marine and Terrestrial Actinomycetes

Chun-Lan Xie [1,2], Jin-Mei Xia [1], Jun-Song Wang [3,*], Dong-Hai Lin [2,*] and Xian-Wen Yang [1,*]

[1] State Key Laboratory Breeding Base of Marine Genetic Resources; Fujian Key Laboratory of Marine Genetic Resources; South China Sea Bio-Resource Exploitation and Utilization Collaborative Innovation Center, Third Institute of Oceanography, State Oceanic Administration; 184 Daxue Road, Xiamen 361005, China; xiechunlanxx@163.com (C. X.); xiajinmei@tio.org.cn (J. X.)

[2] College of Chemistry and Chemical Engineering, the Key Laboratory for Chemical Biology of Fujian Province, MOE Key Laboratory of Spectrochemical Analysis & Instrumentation, Xiamen University, 422 Siming South Road, Xiamen 361005, China

[3] Center for Molecular Metabolism, School of Environmental and Biological Engineering, Nanjing University of Science and Technology, 200 Xiaolingwei Street, Nanjing 210094, China

[#] These authors contributed equally to this work.

* Correspondence: yangxianwen@tio.org.cn (X. Y.), Tel.: +86-592-219-5319； wang.junsong@gmail.com (J. W.), Tel.: +86-25- 8431-5512; dhlin@xmu.edu.cn (D. L.), Tel.: +86-592-218-6078

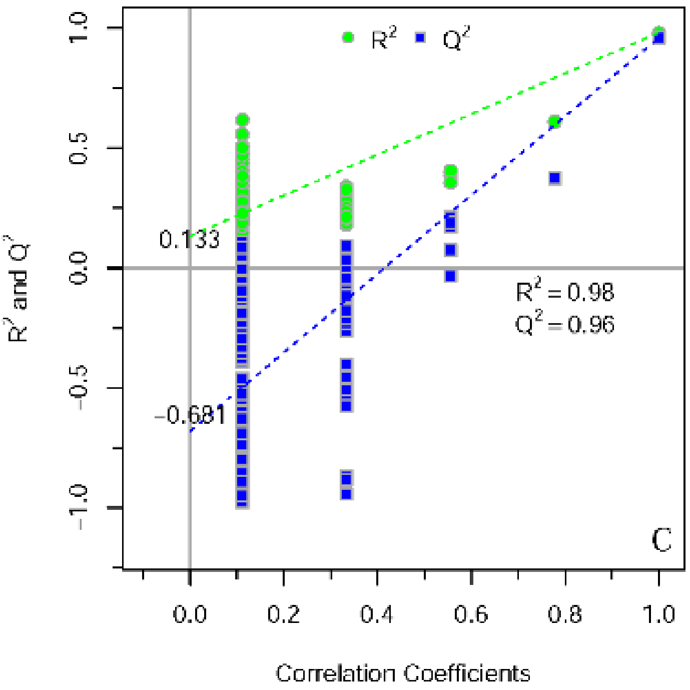

Figure S1. PLS-DA scatter plots of statistical validation obtained by 200 times permutation test. ^{1}H NMR data of intracellular metabolites for four *Nesterenkonia flava* in medium M3.

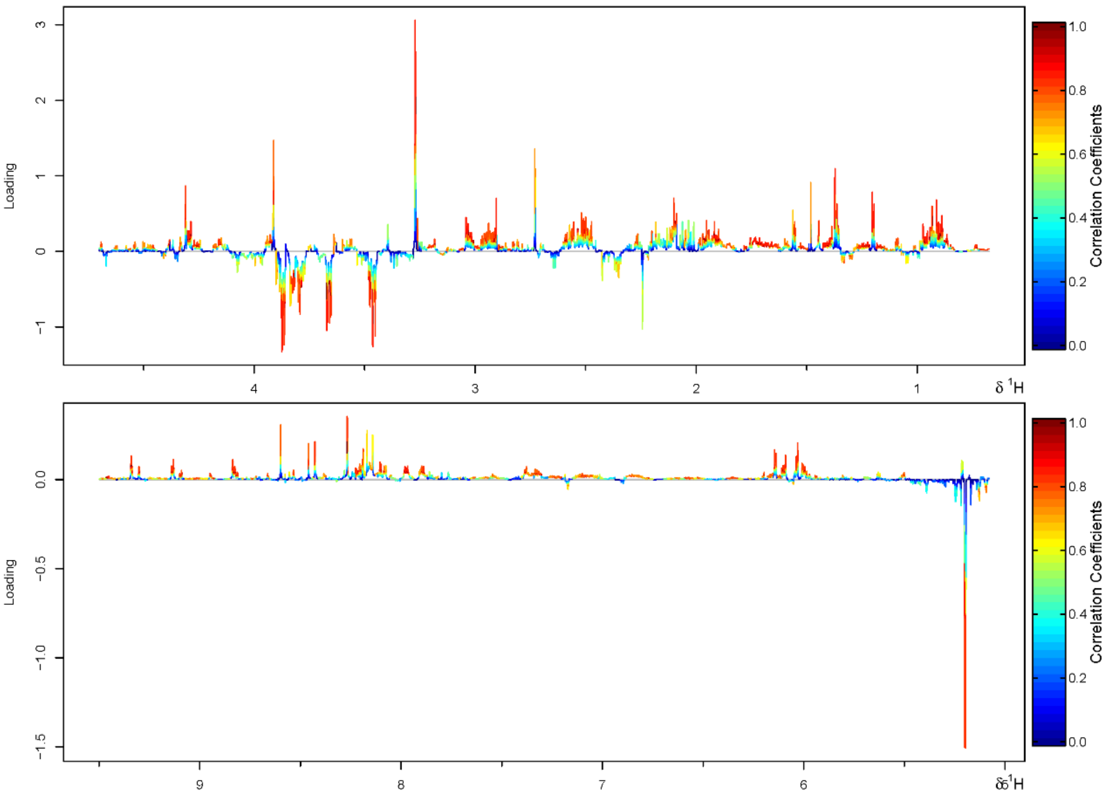

Figure S2. Color-coded loading plot from PLS-DA of ^{1}H NMR data for four strains of *N. flava* cultivated in medium M1.

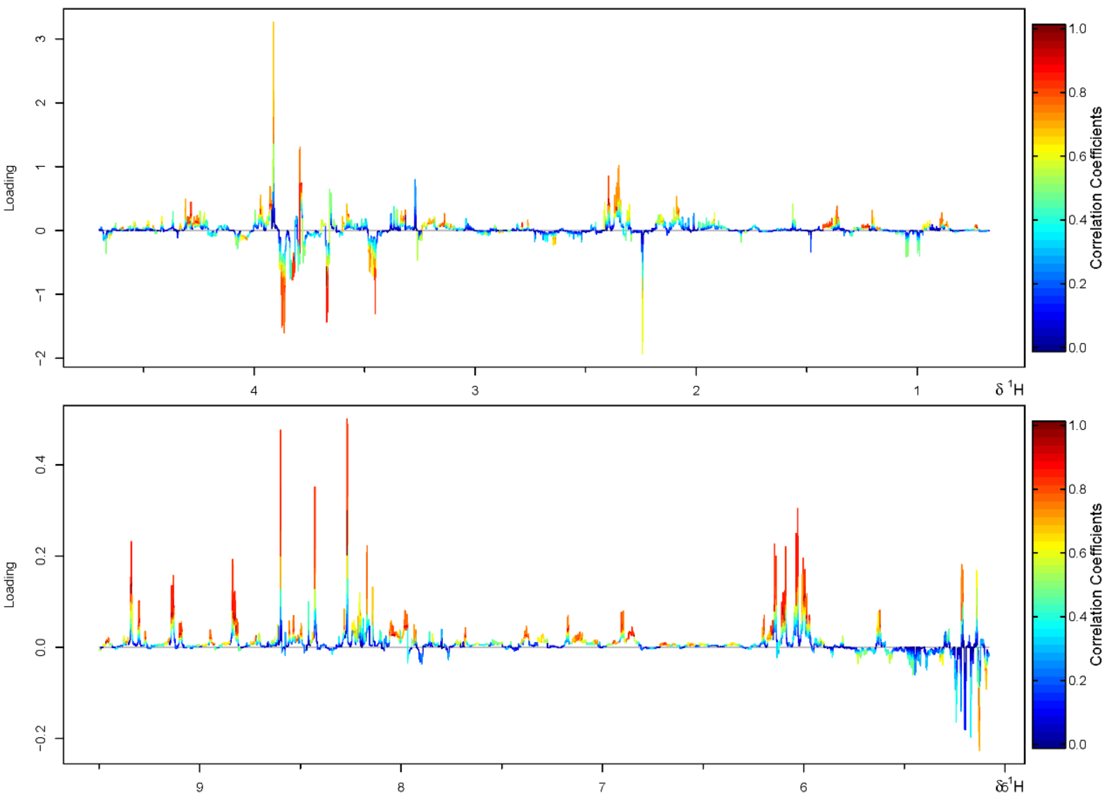

Figure S3. Color-coded loading plot from PLS-DA of ^{1}H NMR data for four strains of *N. flava* cultivated in medium M2.

Table S1. ^{1}H NMR assignments of intracellular metabolites from the marine *Nesterenkonia flava* strain 1K00610 (**Marine-3**) and the terrestrial strain 1A10663 (**Land**) in medium M3. The No. of the metabolites were in accordance with Figure **4**.

No.	Metabolite	Interval (ppm)			
**1**	Pantothenate	0.86-0.92			
**2**	Leucine	0.92-0.95	1.66-1.70		
**3**	Valine	0.99-1.01	1.04-1.06		
**4**	Isoleucine	1.01-1.02			
**5**	3-Hydroxybutyrate	1.19-1.21			
**6**	Fucose	1.22-1.24			
**7**	Threonine	1.33-1.35			
**8**	Alanine	1.48-1.50			
**9**	Lysine	1.70-1.75	1.89-1.90		
**10**	Putrescine	1.75-1.78			
**11**	Thymidine	1.88-1.89			
**12**	Acetic acid	1.91-1.92			
**13**	Acetamide	1.98-1.99			
**14**	*N*-Acetylglucosamine	2.03-2.04			
**15**	*N*-Acetylcysteine	2.04-2.05			
**16**	Glutamate	2.05-2.09	2.11-2.16	2.33-2.37	3.75-3.78
**17**	*p*-Cresol	2.24-2.25			
**18**	Pyruvate	2.37-2.38			
**19**	Succinate	2.39-2.40			
**20**	Methylamine	2.64-2.65			
**21**	Carnosine	2.65-2.70			
**22**	Dimethylamine	2.70-2.72			
**23**	Sarcosine	2.72-2.74			
**24**	Methylguanidine	2.81-2.82			
**25**	Trimethylamine	2.90-2.91			
**26**	Creatine	3.02-3.03			
**27**	Creatine phosphate	3.03-3.04			
**28**	Choline	3.18-3.19			
**29**	Betaine	3.23-3.24			
**30**	Taurine	3.24-3.26			
**31**	Mannose	3.36-3.39	3.57-3.58		
**32**	Maltose	3.40-3.44	3.58-3.64		
**33**	Homogentisate	3.46-3.47			
**34**	Sucrose	3.47-3.50			
**35**	Arabinose	3.50-3.52	4.51-4.53		
**36**	Glycine	3.56-3.57			
**37**	Indole-3-acetate	3.65-3.66			
**38**	Ethylene glycol	3.66-3.67			
**39**	Galactitol	3.68-3.69	3.69-3.71		
**40**	Mannitol	3.85-3.89			
**41**	Vanillate	3.90-3.92			
**42**	Creatinine	4.02-4.04			
**43**	Cytidine	4.12-4.13	4.29-4.31		
**44**	Dihydroxyacetone	4.42-4.43			
**45**	*N*-Acetyl-D-glucosamine	5.19-5.21			
**46**	Glucose	5.23-5.26			
**47**	UDP-glucose	5.96-5.99			
**48**	NAD+	6.02-6.05	6.08-6.12	8.20-8.21	8.42-8.42
**49**	Cholate	7.25-7.35			
**50**	NADH	8.23-8.24	8.44-8.45		

Table S2. List of significant metabolites from PLS-DA (medium M3) which were responsible for the differentiation of marine and terrestrial strains. They were in accordance with Figure **3**. The changing folds (marine relative to terrestrial) and *p*-values were from univariate analysis. The 27 metabolites in bold were characteristic metabolites.

No.	Metabolites abundant in terrestrial strains	Folds	*p*-value	No.	Metabolites abundant in marine strains	Folds	*p*-value
**1**	**Fucose**	0.11	<0.001	**1**	***p*-Cresol**	5.38	<0.001
**2**	**Cholate**	0.20	<0.001	**2**	**Alanine**	4.81	<0.001
**3**	**Pantothenate**	0.23	<0.001	**3**	**Mannose**	4.50	<0.001
**4**	**Leucine**	0.25	<0.001	**4**	**Vanillate**	3.56	0.001
**5**	**Trimethylamine**	0.26	<0.001	**5**	***N*-Acetylcysteine**	3.12	0.024
**6**	**Pyruvate**	0.31	0.002	**6**	***N*-Acetylglucosamine**	2.85	0.018
**7**	**Galactitol**	0.31	<0.001	**7**	**Carnosine**	2.74	0.001
**8**	**NAD^+**	0.42	0.004	**8**	**Sucrose**	2.21	<0.001
**9**	**Lysine**	0.44	<0.001	**9**	**NADH**	1.94	<0.001
**10**	**Glucose**	0.44	0.001	**10**	**Valine**	1.89	<0.001
**11**	**$NADP^+$**	0.45	0.025	**11**	**Methylguanidine**	1.66	0.001
**12**	**3-Hydroxybutyrate**	0.45	<0.001	**12**	**Arabinose**	1.59	0.001
**13**	**Mannitol**	0.52	<0.001	**13**	Sarcosine	1.09	0.211
**14**	**N-Acetyl-D-glucosamine**	0.56	<0.001				
**15**	**UDP-glucose**	0.60	<0.001				
**16**	Indole-3-acetate	0.67	0.211				
**17**	Glutamate	0.70	<0.001				
